# Efficient Degradation of Tetracycline by Peroxymonosulfate Activated with Ni-Co Bimetallic Oxide Derived from Bimetallic Oxalate

**DOI:** 10.3390/toxics12110816

**Published:** 2024-11-14

**Authors:** Qi Zhang, Mingling Yu, Hang Liu, Jin Tang, Xiaolong Yu, Haochuan Wu, Ling Jin, Jianteng Sun

**Affiliations:** 1 School of Environmental Science and Engineering, Guangdong University of Petrochemical Technology, Maoming 525000, China; 2 School of Housing, Building and Planning, Universiti Sains Malaysia, George Town 11800, Pulau Pinang, Malaysia; 3 Department of Civil and Environmental Engineering, The Hong Kong Polytechnic University, Hung Hom, Kowloon 999077, Hong Kong; 24046568g@connect.polyu.hk (M.Y.);

**Keywords:** NiCo_2_O_4_, heterogeneous catalyst, tetracycline, organic pollutants

## Abstract

In this work, NiCo_2_O_4_ was synthesized from bimetallic oxalate and utilized as a heterogeneous catalyst to active peroxymonosulfate (PMS) for the degradation of tetracycline (TC). The degradation efficiency of TC (30 mg/L) in the NiCo_2_O_4_ + PMS system reached 92.4%, with NiCo_2_O_4_ exhibiting satisfactory reusability, stability, and applicability. Radical trapping test and electron paramagnetic resonance (EPR) results indicated that SO_4_^•−^, •OH, O_2_^•−^, and ^1^O_2_ were the dominating reactive oxygen species (ROS) for TC degradation in the NiCo_2_O_4_ + PMS system. Seven intermediates were identified, and their degradation pathways were proposed. Toxicity assessment using T.E.S.T software (its version is 5.1.1.0) revealed that the identified intermediates had lower toxicity compared to intact TC. A rice seed germination test further confirmed that the NiCo_2_O_4_ + PMS system effectively degraded TC into low-toxicity or non-toxic products. In conclusion, NiCo_2_O_4_ shows promise as a safe and efficient catalyst in advanced oxidation processes (AOPs) for the degradation of organic pollutants.

## 1. Introduction

Antibiotics have been widely applied in human or animal medicine because of their powerful antimicrobial capacity and low cost. However, excess antibiotics might be discharged into the aquatic environment via excretion [1], which will bring new environmental problems. Tetracycline, a typical antibiotic, could generate risks to human health and aquatic life due to its metabolism-resistance and toxicity [2,3]. Recent reports have also indicated that TC cannot be effectively removed by traditional technologies, such as biological degradation [4], adsorption [5], and so on. Therefore, due to the refractory biodegradability of TC, it is urgent to find an effective approach to eliminate TC form various water matrices.

Advanced oxidation processes (AOPs) are regarded as being among the most effective technologies for TC control in the aquatic environment [6,7,8]. In particular, peroxymonosulfate (PMS)-based AOPs are becoming increasingly popular among researchers both domestically and internationally [9,10,11] because their asymmetrical structure is easier to activate. PMS-based AOPs exert more advantages in eliminating refractory organic contaminants such as broad pH ranges, low cost, high efficiency, etc. [12,13]. Multiple reactive oxygen species (ROS), including superoxide radical (O_2_^•−^), hydroxyl radical (•OH), sulfate radical (SO_4_^•−^), and singlet oxygen (^1^O_2_), are formed with this technology, which possess a high redox potential and are thus conducive to the degradation of hazardous pollutants [14].

There are many methods by which PMS activation can produce these ROS, such as thermal activation [15], ultrasonic activation [16], UV irradiation [17], and chemical heterogeneous activation [18]. Compared with thermal ultrasonic and UV activation, which are accompanied by excessive energy consumption, chemical heterogeneous activation should be encouraged due to its easy operation conditions, low cost, and high catalytic performance [19,20]. It is reported that transition metal catalysts (Fe, Cu, Mn, and Co) can be applied in PMS-assisted AOPs [21,22,23]. Among them, due to the high redox potential of Co^3+^/Co^2+^ (1.92 V) [24], Co-based catalysts have been proven to be the most efficient catalysts for the removal of organic contaminants via PMS activation [25]. However, cobalt ions releasing with high toxicity will bring new environmental problems.

To overcome this shortcoming of Cobalt-based catalysts, the design and preparation of bimetallic oxides (such as FeCo_2_O_4_, CuCo_2_O_4_, and NiCo_2_O_4_) has been recognized as a promising solution [26,27,28]. Nickel-based materials are also often used to treat organic pollutants in water bodies [29,30]. NiCo_2_O_4_, as a typical bimetallic Co-based catalyst, has high stability and multiple convertible valence states and is considered one of the potential candidates for activating PMS [31]. In particular, the multiple convertible valence states of Co and Ni make mean that they exhibit excellent catalytic properties. However, researchers have invested more effort into the study of the electrochemical properties of NiCo_2_O_4_ [32,33,34] than into the study of its catalytic properties.

In this work, NiCo_2_O_4_ was prepared via the oxalate precipitation method and employed as a heterogeneous catalyst for PMS activation to degrade TC in an aqueous solution. The structure, states, and surface groups were investigated, respectively. The TC degradation experiment was executed in a NiCo_2_O_4_ + PMS system to confirm the catalytic performance of NiCo_2_O_4_. At the same time, the influence of experimental factors (initial pH, NiCo_2_O_4_ dosages, PMS dosages, and TC concentration) on tetracycline degradation was also researched. The cyclic experiment and characterization of fresh and used materials were carried out to reveal the stability. The scavenger experiment and EPR technique were used to identify the ROS in a NiCo_2_O_4_ + PMS system. Furthermore, the degradation products were identified, and possible degradation pathways were proposed. The T.E.S.T software and rice seed germination test were employed to predict and prove the toxicity of products, respectively. Therefore, the aims of this work are the follows: (1) preparing the high-catalytic-activity NiCo_2_O_4_; (2) investigating the degradation efficiency of TC and possible degradation pathways in the NiCo_2_O_4_ + PMS system; (3) detecting whether TC can detoxify in a NiCo_2_O_4_ + PMS system.

## 2. Experimental Section

### 2.1. Chemicals

CoCl_2_·6H_2_O (AR), oxalic acid (H_2_C_2_O_4_·2H_2_O, AR, ≥99.5%), potassium monopersulfate triple salt (PMS, KHSO_5_·0.5KHSO_4_·0.5K_2_SO_4_), furfuryl alcohol (FFA, AR), tert-butanol (TBA, AR), p-Benzoquinone (*p*-BQ, 99%), tetracycline hydrochloride (TC), and ethanol (EtOH, ≥99.8%) were purchased from Aladdin Industrial Co. Ltd., Shanghai, China. NiCl_2_·6H_2_O (AR, ≥98%) was supplied by the Guangzhou Chemical Reagent Factory, Guangzhou, China.

### 2.2. Preparation of NiCo_2_O_4_

The NiCo_2_O_4_ was prepared via the oxalate precipitation method and referenced the synthesis method of CuCo_2_O_4_ [35]. First, 0.01 mol NiCl_2_·6H_2_O and 0.02 mol CoCl_2_·6H_2_O were added into 50 mL deionized water. This solution was marked as A. Then, 0.03 mol H_2_C_2_O_4_ was added into 50 mL deionized water, which was marked as B. Third, solution B was added to solution A, drop by drop, and stirred for 30 min. After cleaning several times, oxalate precipitates were obtained through vacuum filtration. Finally, the oxalate precipitates were calcined in a Muffle furnace (400 °C, 2 h), and the obtained material was marked as NiCo_2_O_4_. Meanwhile, individual cobalt oxides and nickel oxides are also prepared from the calcinations of oxalate precipitates, while 0.03 mol CoCl_2_·6H_2_O or 0.03 mol NiCl_2_·6H_2_O was used.

### 2.3. Characterization of NiCo_2_O_4_

The crystalline phase of the materials was identified via a powder X-ray diffractometer (XRD, Ultima IV, Rigaku, Tokyo, Japan) with wide-angle scanning range of 10–80°. The surface group of the materials was detected via Fourier transform infrared (FTIR) spectra (TENSOR 27, Bruker, Germany). The morphology and elements of the catalyst were detected via scanning electron microscopy coupled to an energy dispersive spectrometer (SEM-EDS) (Bruker, Germany). The Brunauer–Emmett–Teller (BET) surface area and pore size distribution of the materials were obtained from a N_2_ adsorption–desorption isotherm measured on a Micromeritics ASAP 2020 Physisorption. Electron paramagnetic resonance (EPR) measurement was conducted on a JES-FA 300 spectrometer (Japan). The surface chemical states of the elements of the materials were identified via X-ray photoelectron spectroscopy (XPS, Thermo Fisher Scientific K-Alpha, Waltham, MA, USA).

### 2.4. Catalytic Oxidation Experiments

TC degradation experiments were operated in a 200 mL beaker at room temperature (30 ± 2 °C). The pH of the TC solution was adjusted by 0.1 M HCl or 0.1 M NaOH. In a batch experiment, a given amount of catalyst was dispersed in 150 mL of 30 mg/L TC solution with stirring (300 rpm). Then, a given amount of PMS was added into the suspension liquid and the degradation experiment was initiated. At specific time intervals, an aliquot of suspension was collected via syringe and separated via a 0.22 μm hydrophilic filter membrane. Finally, the detection method of TC and its degradation products were referred to in a previous work [36].

### 2.5. Toxicity Prediction of TC Intermediate Products

The toxicity assessment of TC and its intermediate products were predicted by T.E.S.T software (its version is 5.1.1.0). By drawing the chemical structure formula of TC and its products, the T.E.S.T software is used to evaluate and calculate the toxicity. Moreover, a rice seeds germination experiment was conducted to verify the predicted results. First, the solution after the degradation reaction was collected via vacuum filtration. Afterwards, 20 rice seeds were cultivated in a culture dish with the pre- or post-reaction solution (5 mL). The germination of rice was observed on the 5th and 12th days, and the length of the rice seedlings was also measured.

## 3. Results and Discussion

### 3.1. Characterization

**Figure 1:**
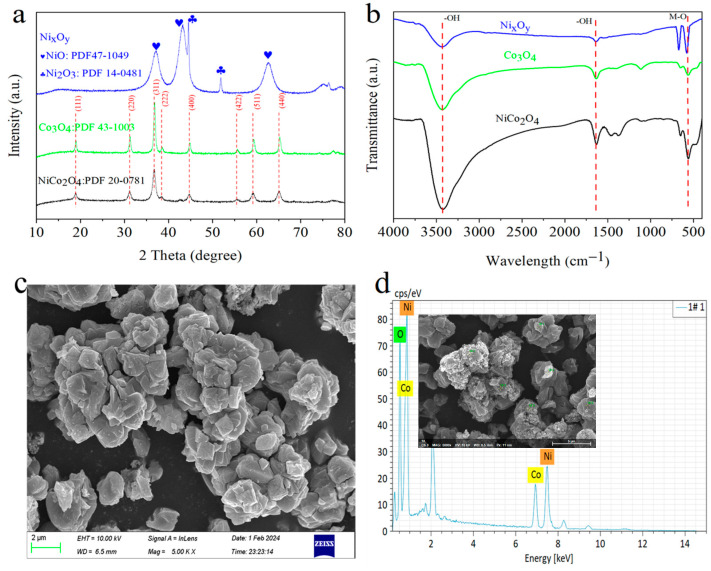
The XRD pattern (**a**), FTIR spectrum (**b**), SEM (**c**), and EDS (**d**) of the obtained NiCo_2_O_4_.

The obtained materials were detected via XRD and FTIR to prove its basic properties, and the results are listed in Figure 1. As shown in Figure 1a, diffraction peaks at 18.91, 31.15, 36.70, 38.40, 44.62, 55.44, 59.08, and 64.98° could be assigned to (111), (220), (311), (222), (400), (422), (511), and (440) facets of NiCo_2_O_4_ (PDF: 20–0781), implying that NiCo_2_O_4_ was successful prepared via the oxalate precipitation method. For the as-synthesized Co_3_O_4_, the diffraction peaks were at 19.00, 31.27, 36.85, 38.55, 44.81, 55.66, 59.35, and 65.23° (PDF: 43–1003), which shifted right by approximately 0.1~0.3° compared to the obtained NiCo_2_O_4_. The diffraction peaks centered at 37.25, 43.28, and 62.88°, which could be indexed to Ni_2_O_3_ (PDF: 14–0481). The diffraction peaks are located at 44.83 and 51.59°, which could be identified as NiO (PDF: 47–1049). Therefore, the obtained Ni_x_O_y_ could consist of Ni_2_O_3_ and NiO based on the XRD information. The chemical structures (or groups) of the obtained catalysts were analyzed via FTIR in the wavenumber range from 400 to 4000 cm^−1^ (Figure 1b). Obviously, the typical transmission peaks are located at approximately 570 cm^−1^, corresponding to the stretching vibration of the M-O bond in Co_3_O_4_, Ni_x_O_y_, and NiCo_2_O_4_. Moreover, the intensity of the M-O bond in NiCo_2_O_4_ was stronger than in Co_3_O_4_ and Ni_x_O_y_, probably because of the existence of Co-O and Ni-O. The weak peak at 1630 cm^−1^ and the broad transmission peak at 3420 cm^−1^ were ascribed to the vibration of the -OH group, which resulted from the adsorbed water on the surface of the catalysts. The SEM images and EDS spectrum of NiCo_2_O_4_ are shown in Figure 1c,d, respectively. The results demonstrated that NiCo_2_O_4_ was composed of agglomerated nanoparticles. Moreover, the catalyst was composed of Ni, Co, and O elements.

The N_2_ adsorption and desorption isotherm was executed to analyze the surface area and pore diameter distribution of catalysts. As shown in Figure S1, according to the classification of the International Union of Pure and Applied Chemistry (IUPAC), the N_2_ adsorption and desorption isotherm of the obtained catalysts exhibited a mixture of type III and IV isotherms at the range of relative pressure P/P_0_ of 0.4–0.9, manifesting the porous property of the material with a large number of mesopores. Three materials showed a H3-type hysteresis loop, demonstrating the existence of slit shape pores [37,38]. As shown in Table S1, the BET surface area was 122.52, 42.42, and 231.69 m^2^/g for NiCo_2_O_4_, Co_3_O_4_, and Ni_x_O_y_, respectively. Additionally, the Barett–Joyner–Halenda (BJH) pore size distribution of the three materials concentrated on 10.35, 22.25, and 5.24 nm for NiCo_2_O_4_, Co_3_O_4_, and Ni_x_O_y_, respectively, which was shown to be useful for the transformation of the contaminant and its by-products in the degradation process.

In this section, XPS was used to analyze the chemical state changes of each element. The full XPS survey scan spectrum of the fresh and used NiCo_2_O_4_ is given in Figure S2. The existence of Ni, Co, and O in the XPS spectrum was consistent with their chemical structures. As shown in Figure S2, the Co 2p spectrum was composed of Co 2p_3/2_ and Co 2p_1/2_, as well as two shakeup satellites. The derived peaks at 779.3 and 794.9 eV represented the spin–orbit doublets properties of Co^II^. The derived peaks at 780.64 and 796.79 eV were identified as Co^III^. These results demonstrated that the cobalt in NiCo_2_O_4_ was in a mixed-valence state, with +2 and +3 states, which might be conducive to activating PMS. The situation of Ni was similar to that of Co, which also existed in divalent and trivalent states. In the O 1 s spectrum, the peaks centered at 529.4, 531.0, and 532.5 eV were assigned to the metal oxygen bond (O-1), the surface hydroxide group (O-2), and the absorbed H_2_O (O-3) [39,40], respectively.

### 3.2. Catalytic Performance of NiCo_2_O_4_

**Figure 2:**
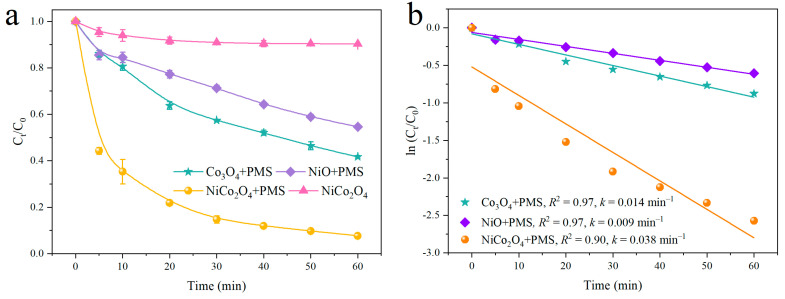
The comparison of different catalysts for TC degradation (**a**) and degradation kinetics (**b**). Conditions (unless otherwise specified in the figures): TC = 30 mg/L; pH = 6.8; temperature = 30 ± 2 °C; PMS = 0.75 mM.

The catalytic performance of NiCo_2_O_4_ was investigated via assessing the degradation efficiency of TC in water media. As shown in Figure 2a, about 10% TC removal was obtained when NiCo_2_O_4_ alone was presented, suggesting that the contribution of TC adsorption toward NiCo_2_O_4_ was negligible during the TC degradation process. With the addition of PMS and different catalysts, PMS could be activated to generate reactive oxygen species (ROS), and the degradation efficiency of TC was different. When Co_3_O_4_ and Ni_x_O_y_ were used as the catalyst, the degradation efficiency of TC was 59 and 46% within 60 min, respectively. Nevertheless, the degradation efficiency of TC in the NiCo_2_O_4_ + PMS system was significantly elevated to 92.4%. These observations demonstrate that NiCo_2_O_4_ possessed better catalytic performance than the other catalysts mentioned above. Furthermore, pseudo-first-order kinetics was used to elucidate the difference in the catalytic performance of these catalysts more intuitively. The corresponding results were concluded in Figure 2b. The *k* values in different systems were obeyed the following order: NiCo_2_O_4_ + PMS (0.038 min^−1^) > Co_3_O_4_ + PMS (0.014 min^−1^) > Ni_x_O_y_ + PMS (0.009 min^−1^), which is consistent with the degradation efficiencies of TC and the decomposition efficiencies of PMS catalyzed by these catalysts (Figure S3). It is shown in Figure S3 that the decomposition efficiency of PMS in the NiCo_2_O_4_ + PMS system could achieve 89% which is distinctly higher than that in the Co_3_O_4_ + PMS and Ni_x_O_y_ + PMS systems. Therefore, combined with these observations, NiCo_2_O_4_ exhibited the highest catalytic activity for TC degradation among the obtained catalysts. In addition, the comparison with the catalytic activity of the similar bimetallic catalyst was concluded in the following (Table S2). In general, the catalytic capacity of the catalysts used in this study was satisfactory.

### 3.3. Identification of ROS and PMS Activation Mechanism

**Figure 3:**
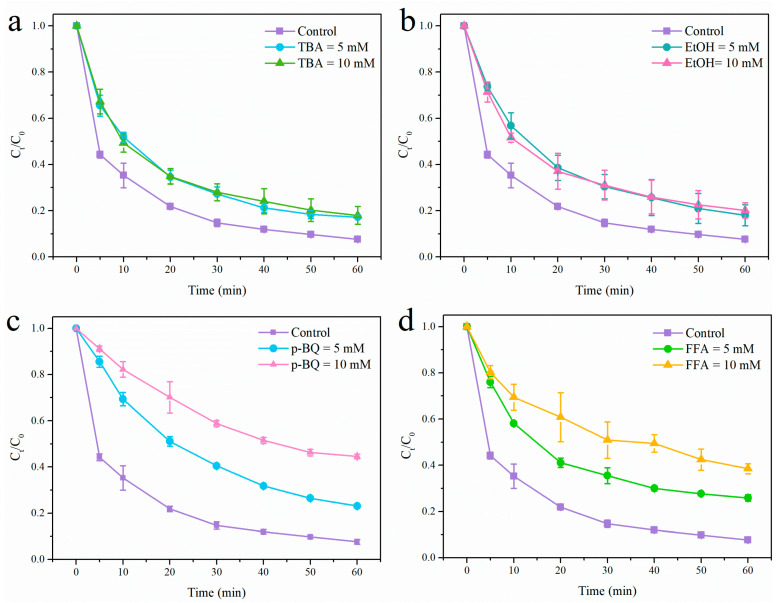
The degradation efficiency of TC in the presence of different scavengers: TBA (**a**); EtOH (**b**); *p*-BQ (**c**); and FFA (**d**). Conditions (unless otherwise specified in the figures): TC = 30 mg/L; pH = 6.8; temperature = 30 ± 2 °C; PMS = 0.75 mM.

As is well known, •OH, SO_4_^•−^, O_2_^•−^, and^−^O_2_ might be the main ROS during the PMS activation process via heterogeneous catalysis reaction. Therefore, in order to identify the main ROS in the NiCo_2_O_4_ + PMS system, a series of quenching experiments were undertaken. In this section, tertiary butanol (TBA), ethyl alcohol (EtOH), *p*-benzoquinone (*p*-BQ)m and furfuryl alcohol (FFA) were used to scavenge •OH, •OH, and SO_4_^•−^, O_2_^•−^, and ^−^O_2_, respectively. As described in Figure 3a, the degradation efficiencies of TC were weakened from 92.4% to 82.8% and 82.1%, with the addition of TBA being 5 and 10 mM, respectively. Likewise, the degradation efficiency of TC was reduced to 82% and 79.9% when EtOH concentration were 5 and 10 mM (Figure 3b), respectively. These observations demonstrated that •OH and SO_4_^•−^ participated in the TC degradation process. Overtly, the addition of *p*-BQ and FFA also reduced the degradation efficiency of TC in this system (Figure 3c,d). Their inhibition for TC degradation stronger than that of TBA and EtOH, declaring that O_2_^•−^ and ^1^O_2_ might be the dominant ROS for TC degradation in comparison to •OH and SO_4_^•−^.

**Figure 4:**
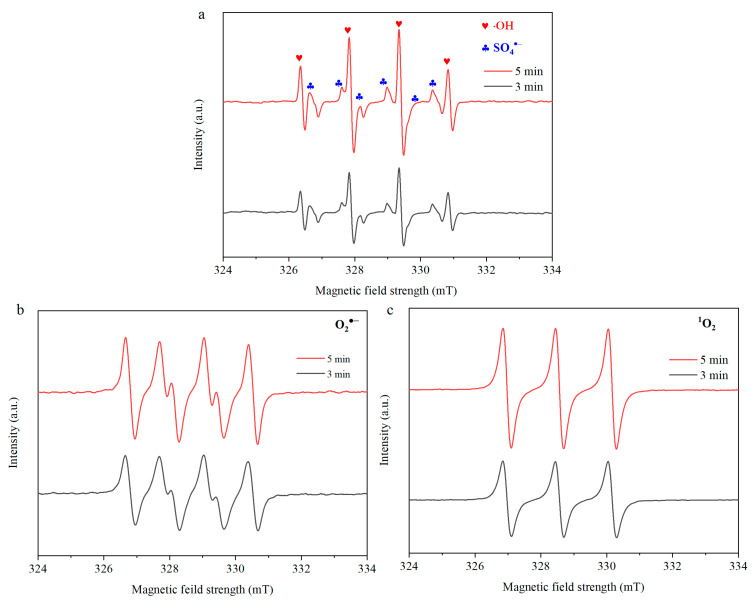
EPR spectrum of the NiCo_2_O_4_ + PMS system with the addition of DMPO or TEMP: (**a**) SO_4_^−^ and OH; (**b**) O_2_^−^; (**c**) ^1^O_2_.

To further confirm the ROS in the NiCo_2_O_4_ + PMS system, EPR detection using DMPO and TEMP as spin-trapping agents was carried out (Figure 4). DMPO is the typical spin-trapping agent to investigate •OH and SO_4_^•−^. As shown in Figure 4a, a strong characteristic peak of DMPO-•OH with a hyperfine splitting of 1:2:2:1 was observed, confirming that •OH was indeed generated in the NiCo_2_O_4_ + PMS system and took part in the degradation of TC. Moreover, the characteristic peaks of the DMPO-SO_4_^•−^ adduct appeared in the vicinity of the DMPO-•OH peaks, suggesting that SO_4_^•−^ was produced during the PMS activation process. Moreover, the signal of O_2_^•−^ was also detected in the NiCo_2_O_4_ + PMS system (Figure 4b). TEMP was employed as the capture agent to identify the existence of ^1^O_2_ involved in the formation of TEMP-O signals. In Figure 4c, it is clear that a strong characteristic signal with a hyperfine splitting of 1:1:1 emerged in the EPR spectrum, offering compelling evidence for the generation of ^1^O_2_ in the NiCo_2_O_4_ + PMS system. Furthermore, the signal intensity of the radicals or non-radicals increased with the reaction time, which was beneficial to the degradation of pollutants and their intermediate products.

Based on the quenching experiment, EPR, and XPS results, the possible PMS activation mechanism in the NiCo_2_O_4_ + PMS system was proposed. Ni and Co sites were identified as the active sites for PMS activation. The characteristic XPS peak of Ni2p and Co 2p had shifted slightly (Figure S2), which indicated that both Co and Ni were involved in the activation of PMS [40]. Moreover, as shown in Figure S2, compared with the fresh NiCo_2_O_4_, the content of O-2 and O-3 of the used NiCo_2_O_4_ increased from 31.8% to 32.8% and from 21.6% to 24.3%, respectively, due to the formation of Ni^II^-OH and Co^III^-OH groups on the surface of NiCo_2_O_4_ [41]. When NiCo_2_O_4_ was added into the TC solution, H_2_O molecules would be adsorbed to the M (Ni^II^ and Co^III^) site of the NiCo_2_O_4_ surface to form M-OH (Ni^II^-OH and Co^III^-OH). The formed Ni^II^-OH and Co^III^-OH would react with the added PMS and generate SO_4_^•−^ for degrading TC. Meanwhile, Ni^II^-OH and Co^III^-OH could be transformed into Ni^III^-OH and Co^II^-OH, respectively. Ni^III^-OH and Co^II^-OH also reacted with PMS, causing their state to return to its previous state. Therefore, the cycles of Ni^II/^Ni^III^ and Co^III/^Co^II^ were formed during the PMS activation process and were conducive to PMS decomposition and radical (such as SO_4_^•−^, •OH, and SO_5_^−^) generation. It should not be ignored that some SO_4_^•−^ could be converted into •OH [42]. H_2_O_2_ would be produced due to the generated •OH, followed by a series of reactions and thus leading to the generation of O_2_^•−^ [43]. On the other hand, ^1^O_2_ could be generated via the reaction between SO_5_^•−^ and PMS [44]. Eventually, the generated radicals and non-radicals would degrade the TC molecule, resulting in the formation of small molecule substances or less harmful products.

### 3.4. Reusability, Stability, and Applicability of NiCo_2_O_4_

**Figure 5:**
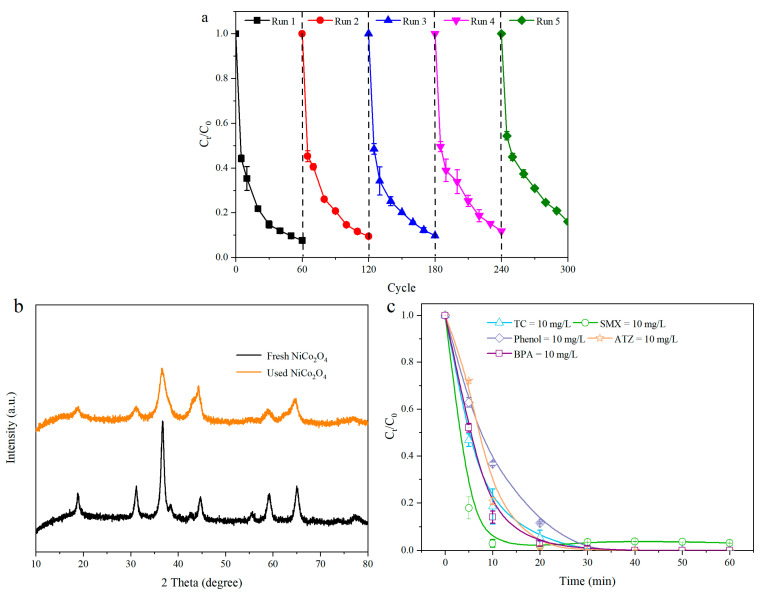
The reusability, stability, and applicability of NiCo_2_O_4_ (**a**). Conditions (unless otherwise specified in the figures): TC = 30 mg/L; pH = 6.8; temperature = 30 ± 2 °C; PMS = 0.75 mM. The XRD of the fresh and used of NiCo_2_O_4_ (**b**). The degradation efficiency of other organic contaminants in the NiCo_2_O_4_ + PMS system (**c**).

The stability of the catalyst was a momentous indicator in the practical application. Figure 5a displays the degradation efficiency of TC in the oxidation process during five cycles catalyzed by NiCo_2_O_4_. The degradation efficiency of TC approached 92.4%, 90.5%, 90.3%, 88.3%, and 84.0% in every cycle. Compared with the first degradation cycle, the degradation efficiency was only reduced by 8.4%. To sum up, the above results confirmed that NiCo_2_O_4_ possessed satisfactory reusability. The intensity of the characteristic peak of the used NiCo_2_O_4_ was weaker than the fresh NiCo_2_O_4_, yet the crystal structure of the used NiCo_2_O_4_ did not change significantly from the XRD spectra (Figure 5b), indicating that this material maintained its stability during the reaction process. In this work, the other typical organic contaminants, such as sulfamethoxazole (SMX), bisphenol A (BPA), atrazine (ATZ), and phenol, were also selected to verify the catalytic performance and applicability of NiCo_2_O_4_. As shown in Figure 5c, it is clear that multiple pollutants, including SMX, phenol, ATZ, and BPA, could almost be degraded in the NiCo_2_O_4_ + PMS system within 60 min, indicating that a combination of PMS with NiCo_2_O_4_ has infinite application potential with respect to various organic pollutants.

### 3.5. Effect of Experimental Factors

**Figure 6:**
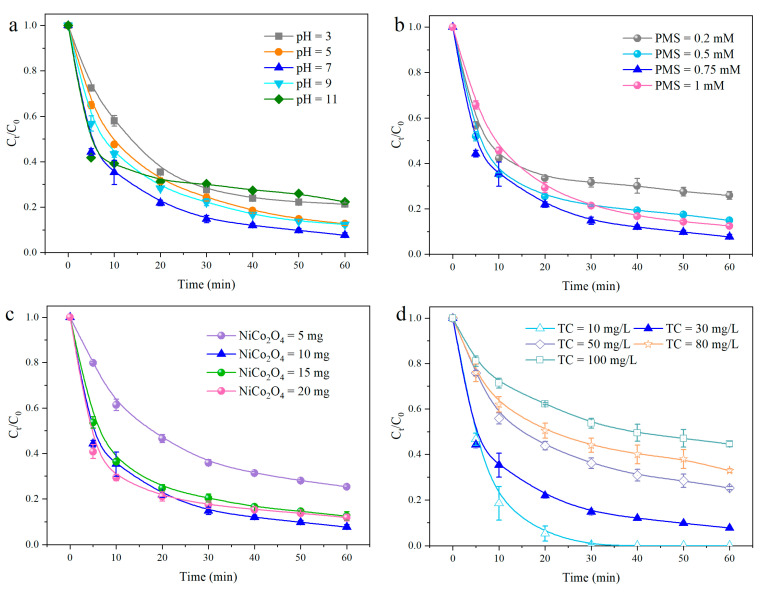
The effect of experimental factors on TC degradation: pH (**a**); PMS concentration (**b**); NiCo_2_O_4_ dosage (**c**); and TC concentration (**d**). Conditions (unless otherwise specified in the figures): TC = 30 mg/L; pH = 6.8; temperature = 30 ± 2 °C; PMS = 0.75 mM.

In this study, the effect of experimental factors on TC degradation in the NiCo_2_O_4_ + PMS system was investigated, including solution pH, PMS concentration, NiCo_2_O_4_ dosage, and initial TC concentration. As is well known, solution pH is a vital factor influencing catalytic performance in a heterogeneous catalytic system. It is clear in Figure 6a that TC degradation was slightly increased with the increase in initial pH from 3 to 7, accompanied by the degradation efficiency of TC varying from 78.7% to 92.4%. However, the degradation efficiency of TC was inhibited from 92.4% to 77.8% with the further increase in initial pH. Generally, the NiCo_2_O_4_ + PMS system exhibited preferable potential and was suitable for complex environments.

The effect of PMS concentration on TC degradation was studied, and the results are depicted in Figure 6b. Apparently, the degradation efficiency of TC was promoted with the increased PMS dosage in the range of 0.2–0.75 mM. As oxidant concentration increased, more ROS could be produced, resulting in more and faster TC degradation. On the other hand, the degradation efficiency of TC was slightly reduced when PMS concentration was 1.0 mM. The self-quenching effect of free radicals under a higher PMS concentration was previously reported [45]. In addition, the generated ROS might be rapidly consumed by the excess oxidants [46], which also affected the degradation efficiency of target contaminant.

The impact of catalyst dosage on the degradation efficiency of TC was also studied, and the result is shown in Figure 6c. After 60 min, the degradation efficiency of TC was enhanced first and then reduced with the increase in catalyst dosage, reaching the maximum value when the catalyst dosage was 10 mg. The increased degradation efficiency might be ascribed to the fact that the increase in NiCo_2_O_4_ dosage could offer more active sites, which is conducive to the activation of PMS and the generation of ROS. Of course, a slight reduction was also observed with the further increase in NiCo_2_O_4_ dosage to 15 and 20 mg owing to a diffusion limitation phenomenon under the excess catalyst in a heterogeneous system [47].

The effect of the initial TC concentration on its degradation is presented in Figure 6d. Obviously, TC degradation was influenced by the changes in the initial concentration from 10 to 100 mg/L, with the corresponding degradation efficiency weakening from nearly 100% to 55.5%. Only constant ROS could be produced in a constant catalyst and oxidant system, so the degradation efficiency of the target pollutant would be affected.

### 3.6. Effect of Co-Existing Anions

The co-existing anions were ubiquitous in the aquatic water environment and reacted with the generated ROS, which could affect the degradation of the target organic pollutant in the PMS activation system. As van be seen in Figure S4, the presence of co-existing anions, such as Cl^−^, NO^3−^, SO_4_^2−^, HCO_3_^−^, and HPO_4_^2−^, resulted in the inhibition of the degradation efficiency of TC. The inhibition effect of Cl^−^ was ascribed to the consumption of radicals in the catalytic system, leading to the production of Cl^•^/ClOH^•^, which has a lower oxidation ability (Equations (1) and (2)) [48]. The concomitant NO_3_^−^ also influenced TC degradation to a slight degree since it trapped SO_4_^•−^ or •OH species and hence formed the corresponding radicals (Equations (3) and (4)) [49]. Comparably, the SO_4_^2−^, HCO_3_^−^, and HPO_4_^2−^ affected the degradation efficiency of TC via their consumption of the produced radicals in this system. Similar results were observed in several PMS activation systems, such as CuO + PMS or Fe_3_O_4_ + PMS [50,51]. Although the degradation efficiency of TC was interfered by other ions, the degradation efficiency was still maintained over 70% in the NiCo_2_O_4_ + PMS system, which indicated that NiCo_2_O_4_, as a catalyst, could also produce a better catalytic effect under complex conditions.

(1) $${\mathrm{Cl}}^{-}+{\mathrm{SO}}_{4}^{•-}\text{→}{\mathrm{Cl}}^{•-}+{\mathrm{SO}}_{4}^{2-}$$

(2) $${\mathrm{Cl}}^{-}+•\mathrm{OH}\text{→}{\mathrm{ClOH}}^{•-}$$

(3) $$NO_{3}^{-}+{\mathrm{SO}}_{4}^{•-}\text{→}{\mathrm{NO}}_{3}^{•}+{\mathrm{SO}}_{4}^{2-}$$

(4) $${\mathrm{NO}}_{3}^{-}+•\mathrm{OH}\text{→}{\mathrm{NO}}_{3}^{•}+{\mathrm{OH}}^{-}$$

### 3.7. Possible Intermediates of TC and Its Toxicity Assessment

The degradation products were detected via ultra-performance liquid chromatography coupled with Q-Exactive Orbitrap mass spectrometry. In this work, seven intermediates were identified, including P I (*m*/*z* 413), P II (*m*/*z* 359), P III (*m*/*z* 337), P IV (*m*/*z* 297), P V (*m*/*z* 223), P VI (*m*/*z* 459), and P VII (*m*/*z* 431), and their MS/MS mass spectra are listed in Figure S5–S11. Based on this information, the possible degradation pathway of TC is proposed in Figure S12. On the one hand, TC was attacked by ROS, resulting in the generation of P I. One phenol group of P I was removed under the attack of ROS; thus, P II was formed [52]. Furthermore, under the continuous attack of ROS, P III, IV, and V were generated during the degradation process. Eventually, some open-ring products were detected during this process. At the same time, similar open-ring phenomena have been found in other PMS systems [53]. On the other hand, TC was directly oxidized by ROS and formed ketone in P VI. In addition, P VI could be further oxidized to P VII via a bi-demethylation reaction. This process has also been found in a previous study [54]. In summary, the detection of the product confirmed that tetracycline was indeed degraded in the system, rather than adsorbed.

**Figure 7:**
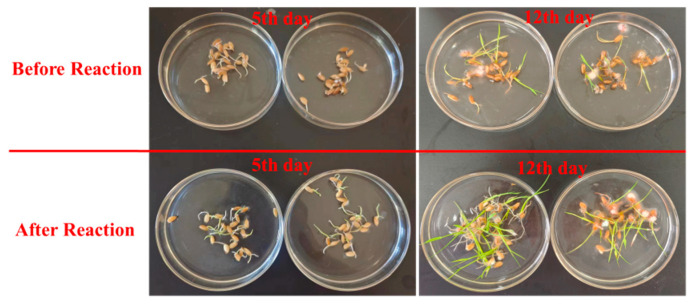
The germination of rice seedlings in before reaction solution and after reaction solution.

In this work, the toxicity assessment of the identified intermediates was predicted by T.E.S.T software, and the results are displayed in Table S3. Obviously, most of the predicted values of these products were higher than those of the parent TC, indicating that TC could be gradually converted into less toxic products under this system. In this work, a rice seed germination experiment was conducted to verify the toxicity assessment of the identified intermediates. These prediction results were also similar to the germination and growth of rice seeds. As shown in Figure 7, there is little difference in the seed germination efficiency (5d) between the pre-reaction and post-reaction solution, yet the rice seedling growth in the post-reaction solution was better than in the pre-reaction solution. Satisfactorily, the growth of rice seedlings in the pre-reaction solution (12d) was significantly superior to that in the post-reaction solution. Some rice seedlings in the pre-reaction solution (12d) gradually turned yellow or even died. After screening and examination, only eight rice seedlings in the pre-reaction solution (12d) were alive in each culture dish, with a survival efficiency of 40%. However, in the post-reaction solution, 17 rice seedlings were alive and growing well in each culture dish, with a survival efficiency of 85%. Additionally, the length of each rice seedling was measured with a ruler and the results are displayed in Figure S13. As shown in Figure S13, the length of a rice seedling in the post-reaction solution was better than that in the pre-reaction solution. The average length of a rice seedling in the post-reaction solution was 3.52 ± 0.78 cm, yet the average length in the pre-reaction solution was just 2.27 ± 0.24 cm. This indicates that TC degradation in the NiCo_2_O_4_ + PMS system is conducive to alleviating its toxicity and reconfirms the results of toxicity prediction attained via T.E.S.T software. Hence, the above observations demonstrated that the NiCo_2_O_4_ + PMS system was suited to the control of organic pollutants.

## 4. Conclusions

In this work, NiCo_2_O_4_ was successfully synthesized and employed to degrade TC via PMS activation. The experimental results indicated that the NiCo_2_O_4_ + PMS system was also suitable for degrading organic pollutants with high efficiency. NiCo_2_O_4_ could be recycled many times and showed good applicability. In the NiCo_2_O_4_ + PMS system, radical (SO_4_^•−^, •OH, and O_2_^•−^) and non-radical (^1^O_2_) played a pivotal role in TC degradation. Multiple degradation products were identified, and their degradation pathways were analyzed. The toxicity of the degradation products was less than that of the original TC according to toxicity prediction software. As to the germination of rice seeds (5th day and 12th day), the germination of rice seeds in the post-reaction solution and the growth of the rice seeds were better when compared to the rice seeds in the pre-reaction solution, indicating that the toxicity of the degradation products was lower. This result was also consistent with the toxicity prediction result. In general, the NiCo_2_O_4_ + PMS system has good application prospects and potential in the treatment of organic pollutants via heterogeneous AOPs. Bimetallic oxide catalysts have potential applications in the field of heterogeneous AOPs.

## Data Availability

All the data and material were shown in the manuscript.

## Supplementary Materials

The following supporting information can be downloaded at: https://www.mdpi.com/article/10.3390/toxics12110816/s1. Figure S1. The N2 adsorption and desorption isotherm (a) and BJH pore diameter distribution (b). Figure S2. The XPS scan spectrum of the fresh and used NiCo2O4. (a) survey, (b) Co 2p, (c) Ni 2p and (d) O 1s. Figure S3. The decomposition efficiency of PMS in different systems. Conditions (Unless otherwise specified in the figures): TC = 30 mg/L, pH = 6.8, Temperature = 30 ± 2 °C, PMS = 0.75 mM. Figure S4. The effect of co-existing anions on TC degradation in NiCo2O4+PMS system. Conditions (Unless otherwise specified in the figures): TC = 30 mg/L, pH = 6.8, Temperature = 30 ± 2 °C, PMS = 0.75 mM. Figure S5. The MS/MS mass spectrum of Product I. Figure S6. The MS/MS mass spectrum of Product II. Figure S7. The MS/MS mass spectrum of Product III. Figure S8. The MS/MS mass spectrum of Product IV. Figure S9. The MS/MS mass spectrum of Product V. Figure S10. The MS/MS mass spectrum of Product VI. Figure S11. The MS/MS mass spectrum of Product VII. Figure S12. The possible degradation pathways of TC in NiCo_2_O_4_+PMS system. The dashed box indicates that the product is not detected [52]. Figure S13. The length of rice seedings in before and after reaction solution. Table S1. The BET surface area (m^2^/g) and BJH pore size distribution (nm) of the obtained catalysts in this work. Table S2. The comparison of the catalytic activity of NiCo_2_O_4_+PMS with other catalysts [55,56,57,58]. Table S3. The toxicity assessment of the identified intermediates was predicted by T.E.S.T software.
