# Supplementary material for: Efficient Degradation of Tetracycline by Peroxymonosulfate Activated with Ni-Co Bimetallic Oxide Derived from Bimetallic Oxalate
Source: Toxics. 2024 Nov 14;12(11):816. doi: 10.3390/toxics12110816 (PMC11598248; doi:10.3390/toxics12110816)
Supplement: Supplementary file 1 [file toxics-12-00816-s001.zip › toxics-3270236-supplementary.pdf]

Supplementary Information

Efficient Degradation of Tetracycline by Peroxymonosulfate Activated with Ni-Co Bimetallic Oxide Derived from Bimetallic Oxalate

Qi Zhang[a,b], Mingling Yu[c], Hang Liu[a], Jin Tang[a,*], Xiaolong Yu[a], Haochuan Wu[a,b], Ling Jin[c], Jianteng Sun[a,*]

[a] School of Environmental Science and Engineering, Guangdong University of Petrochemical Technology, Maoming, Guangdong, 525000, China

[b] School of Housing, Building and Planning, Universiti Sains Malaysia, George Town 11800, Pulau Pinang, Malaysia

[c] Department of Civil and Environmental Engineering, The Hong Kong Polytechnic University, Hung Hom, Kowloon, 999077, Hong Kong

* Corresponding author.

E-mail address: jintang117@foxmail.com (J. Tang); sunjianteng@zju.edu.cn (J. Sun)

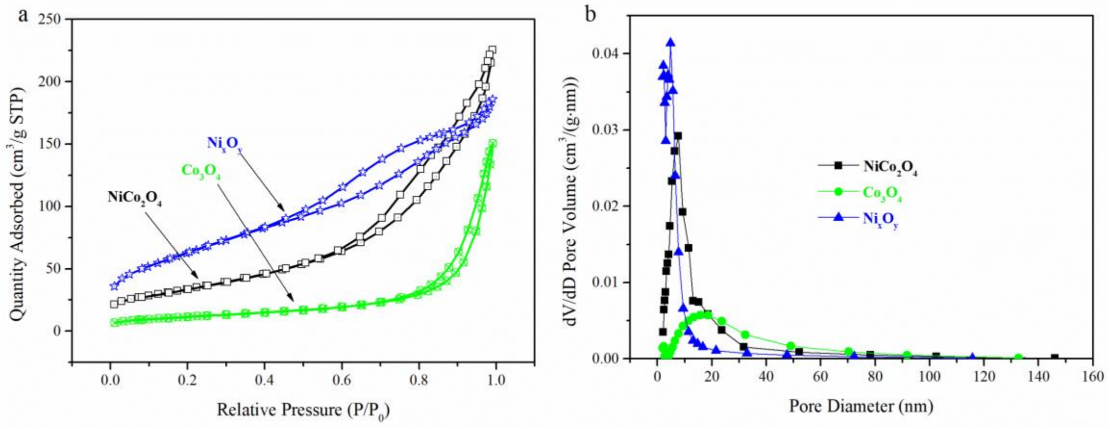

Figure S1 The N_2 adsorption and desorption isotherm (a) and BJH pore diameter distribution (b).

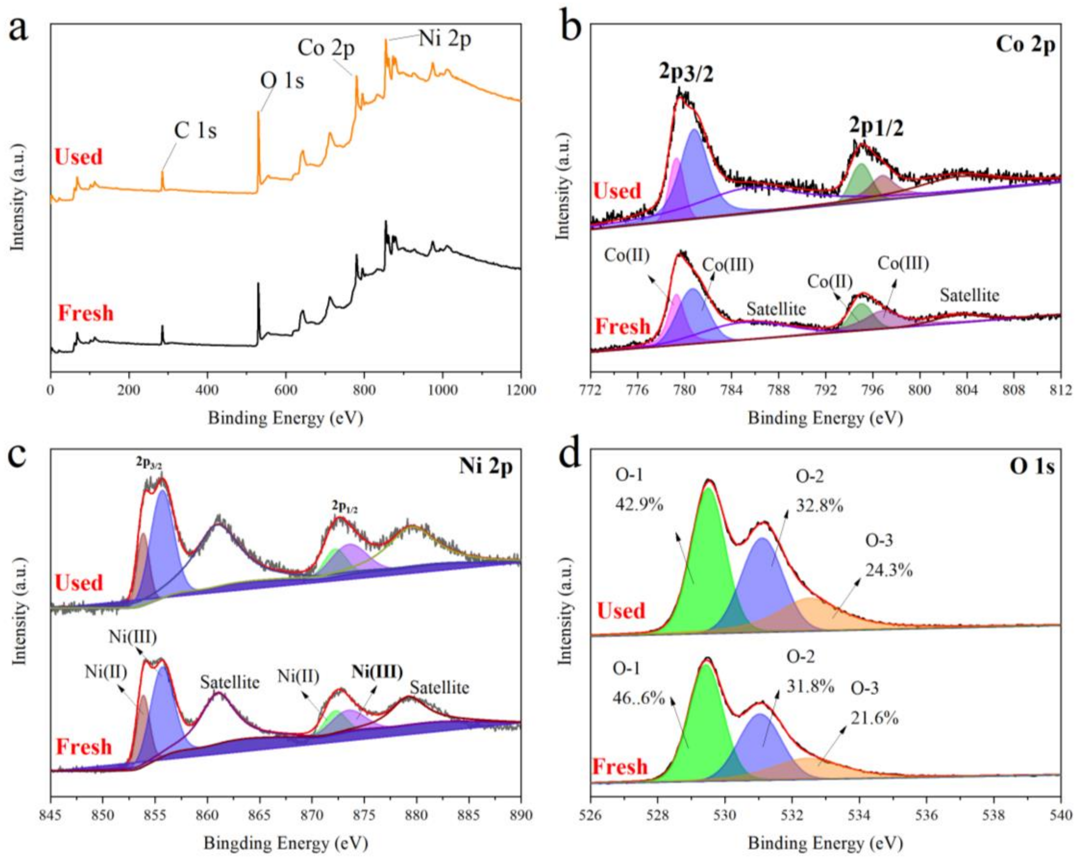

Figure S2 The XPS scan spectrum of the fresh and used NiCo2O4. (a) survey, (b) Co 2p, (c) Ni 2p and (d) O 1s.

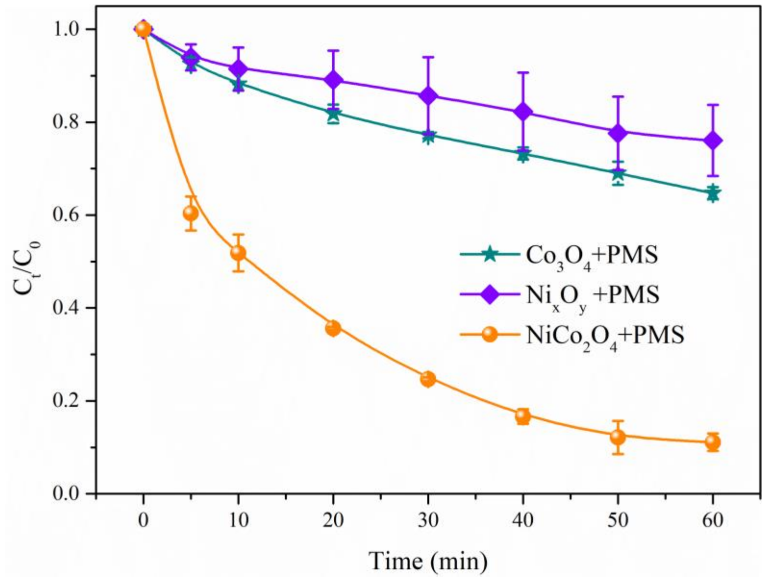

Figure S3. The decomposition efficiency of PMS in different systems. Conditions (Unless otherwise specified in the figures): TC = 30 mg/L, pH = 6.8, Temperature = 30±2 °C, PMS = 0.75 mM.

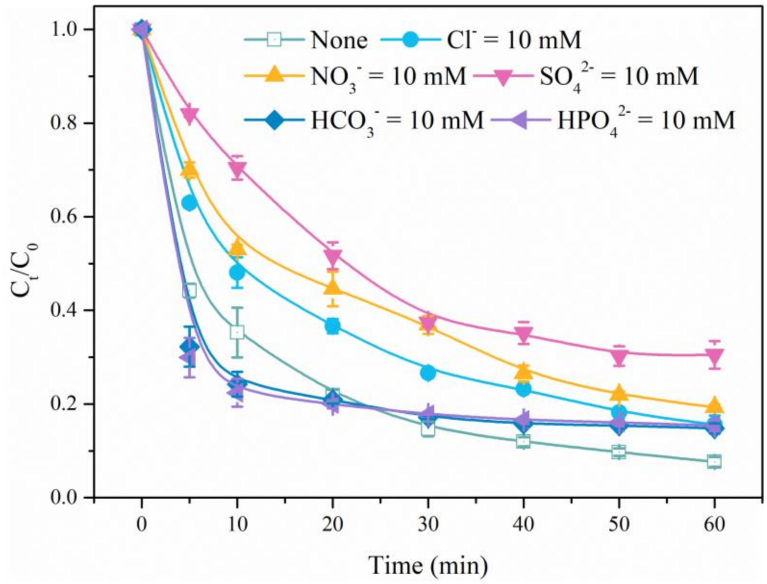

Figure S4. The effect of co-existing anions on TC degradation in $NiCo_2O_4$+PMS system. Conditions (Unless otherwise specified in the figures): TC = 30 mg/L, pH = 6.8, Temperature = 30±2 °C, PMS = 0.75 mM.

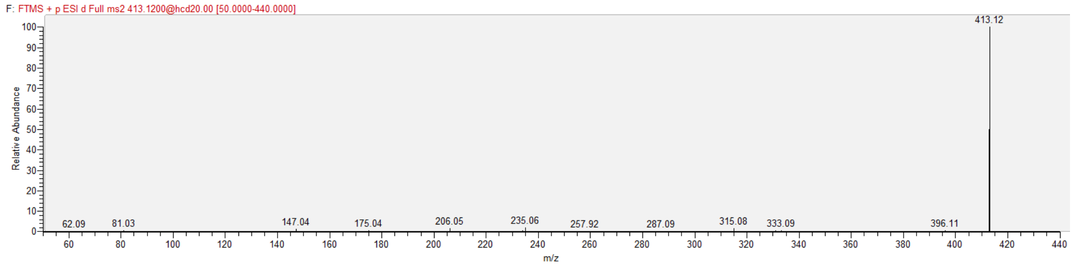

Figure S5. The MS/MS mass spectrum of Product I

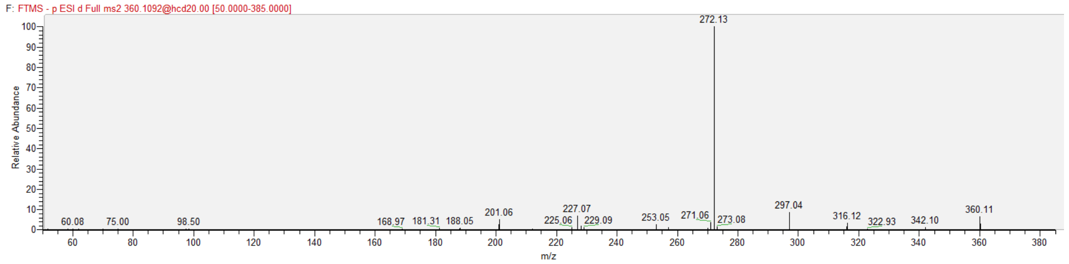

Figure S6. The MS/MS mass spectrum of Product II.

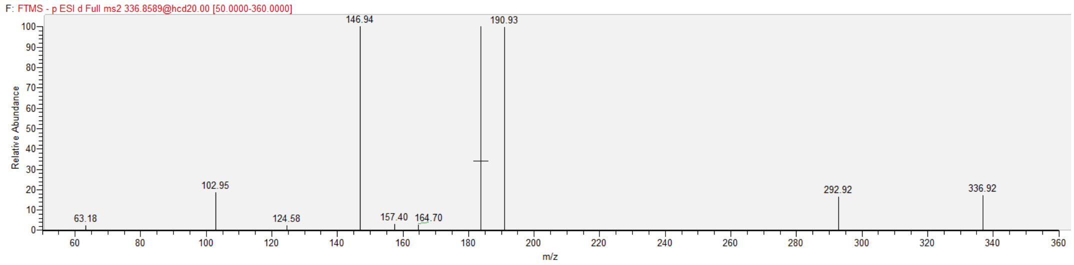

Figure S7. The MS/MS mass spectrum of Product III.

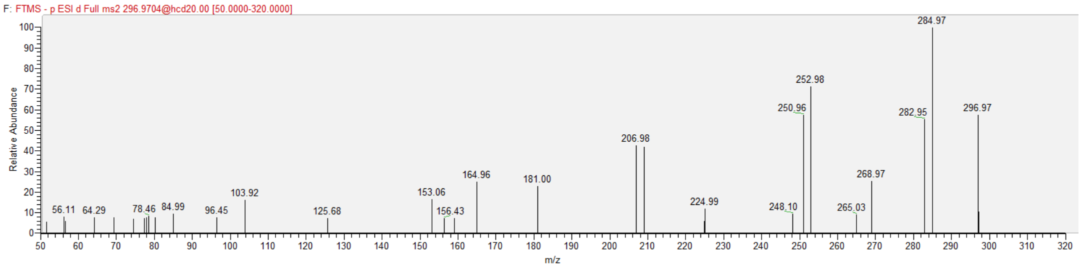

Figure S8. The MS/MS mass spectrum of Product IV.

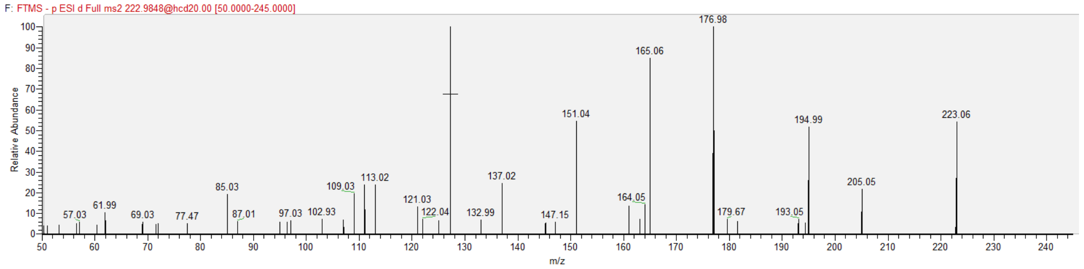

Figure S9. The MS/MS mass spectrum of Product V.

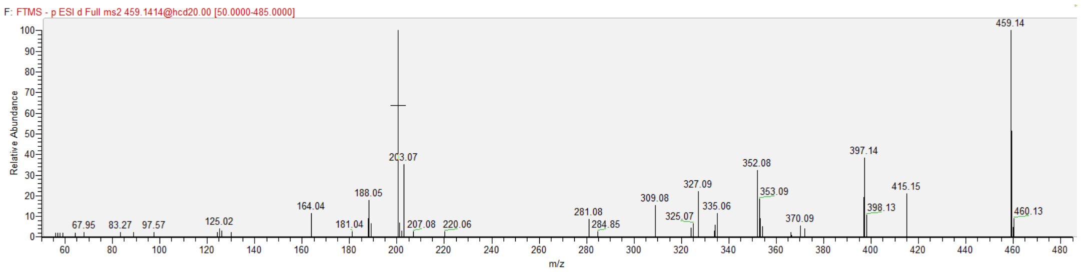

Figure S10. The MS/MS mass spectrum of Product VI.

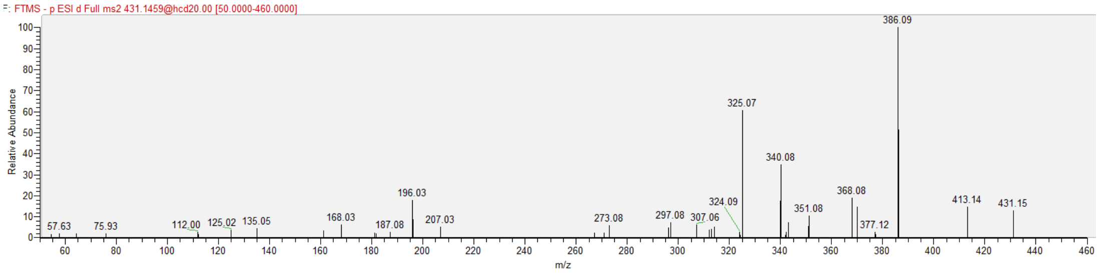

Figure S11. The MS/MS mass spectrum of Product VII.

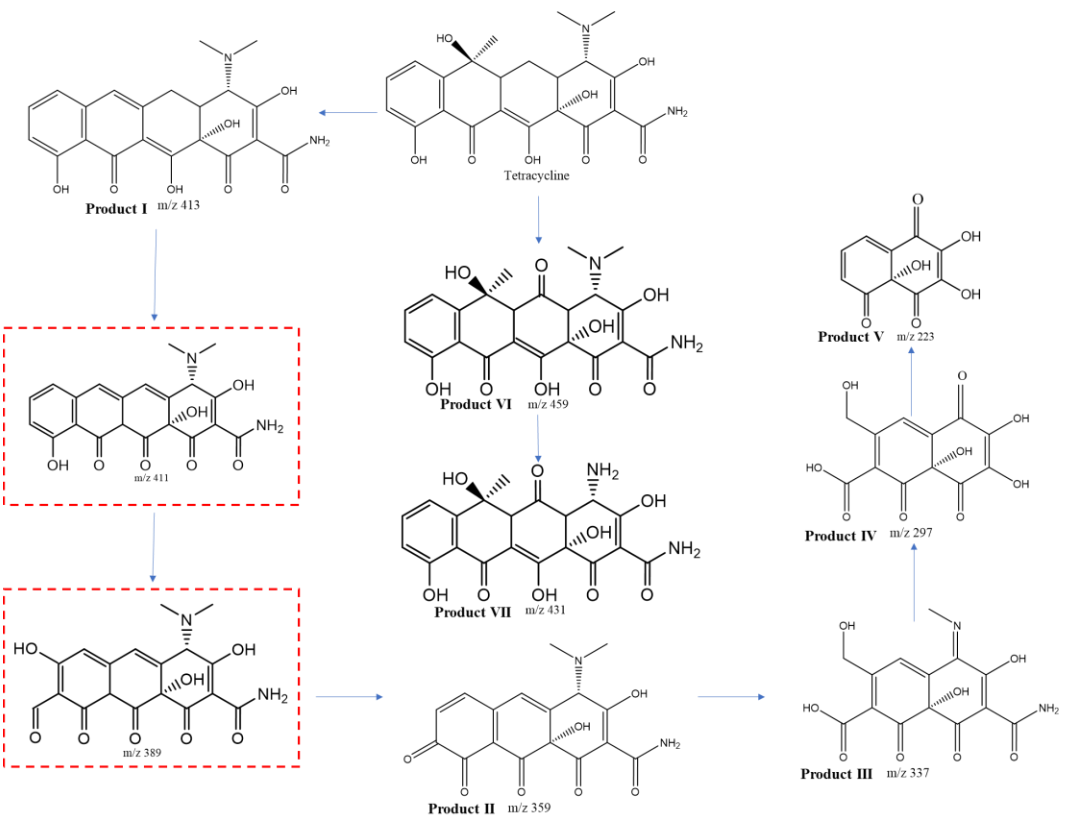

Figure S12. The possible degradation pathways of TC in $NiCo_2O_4$+PMS system. The dashed box indicates that the product is not detected [1].

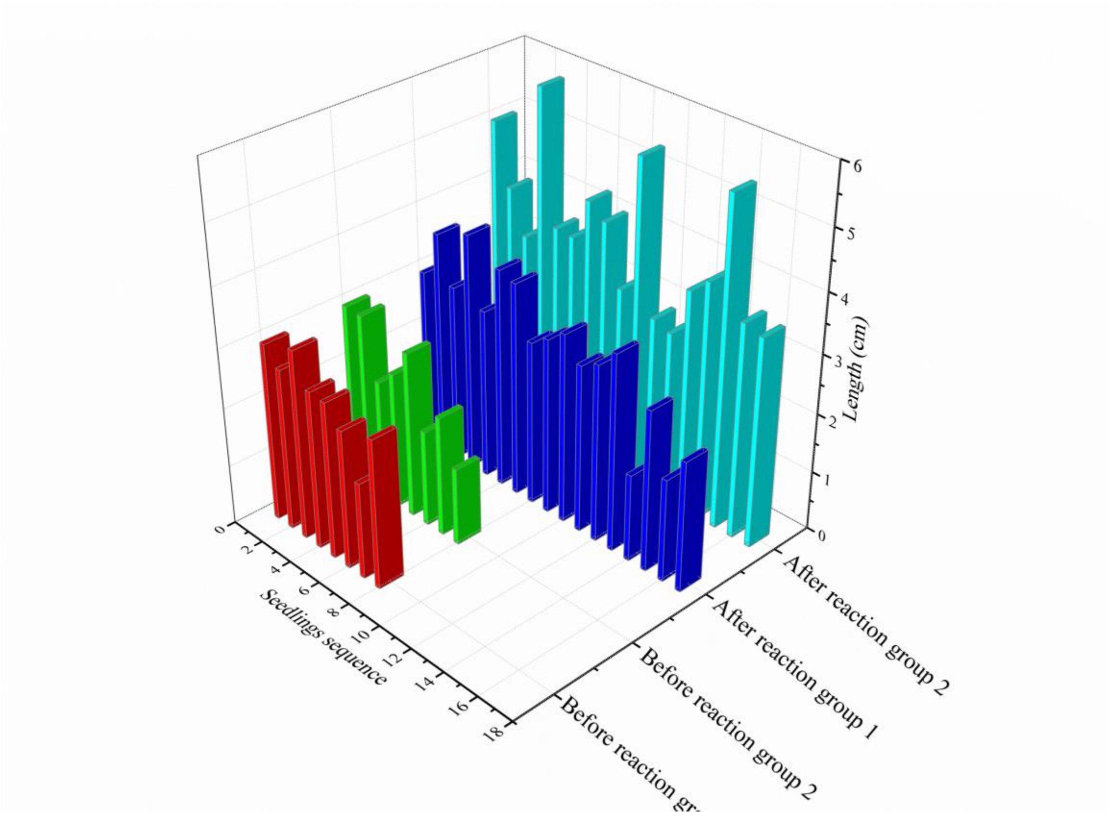

Figure S13. The length of rice seedings in before and after reaction solution.

Table S1. The BET surface area (m^2/g) and BJH pore size distribution (nm) of the obtained catalysts in this work.

Catalsyts	BET surface area (m^2/g)	BJH pore size distribution (nm)
$NiCo_2O_4$	122.52	10.35
Co_3O_4	42.42	22.25
Ni_xO_y	231.69	5.24

Table S2. The comparison of the catalytic activity of $NiCo_2O_4$+PMS with other catalysts.

System	Antibiotics	Degradation efficiency	References
$CoFe_2O_4$ NCs+PMS	chloramphenicol (10 mg/L)	100% (120min)	[2]
$CoFe_2O_4$/OSC+PMS	Norfloxacin (30μM)	90.8% (60 min)	[3]
$MnCo_2O_4$+light+PMS	Tetracycline (20mg/L)	80% (12min)	[4]
$NiCo_2O_4$/g-C_3N_4-N_{vac} +PMS	Tetracycline (20mg/L)	84.4% (30min)	[5]
$NiCo_2O_4$+PMS	Tetracycline (30mg/L)	92.4% (60min)	This work

Table S3. The toxicity assessment of the identified intermediates was predicted by T.E.X.T software.

Name	Chemical Structure	Endpoint	Predicted
**TC**	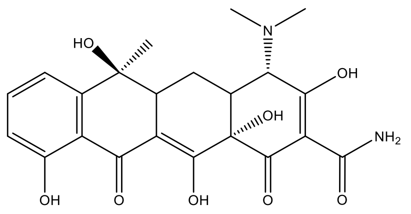	Ora Rat LD50 (mg/kg)	1068.64
		Fathead minnow LC50 (96 hr) (mg/L)	0.90
		Daphnia magna LC50 (48hr) (mg/L)	8.73
**P I**	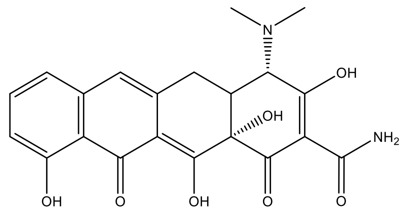 m/z 413	Ora Rat LD50 (mg/kg)	1329.16
		Fathead minnow LC50 (96 hr) (mg/L)	0.16
		Daphnia magna LC50 (48hr) (mg/L)	1.81
**P II**	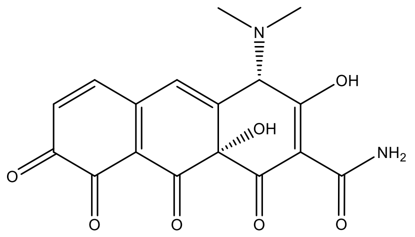 m/z 359	Ora Rat LD50 (mg/kg)	1955.03
		Fathead minnow LC50 (96 hr) (mg/L)	1.88
		Daphnia magna LC50 (48hr) (mg/L)	22.76
**P III**	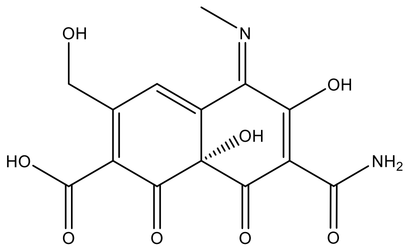 m/z 337	Ora Rat LD50 (mg/kg)	3233.56
		Fathead minnow LC50 (96 hr) (mg/L)	3.64
		Daphnia magna LC50 (48hr) (mg/L)	47.62
**P IV**	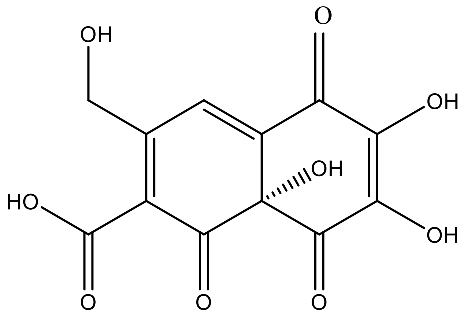 m/z 297	Ora Rat LD50 (mg/kg)	3885.46
		Fathead minnow LC50 (96 hr) (mg/L)	3.86
		Daphnia magna LC50 (48hr) (mg/L)	78.94

**P V**	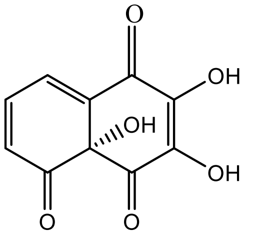 m/z 223	Ora Rat LD50 (mg/kg)<br>Fathead minnow LC50 (96 hr) (mg/L)<br>Daphnia magna LC50 (48hr) (mg/L)	1089.08<br>119.98<br>64.73
**P VI**	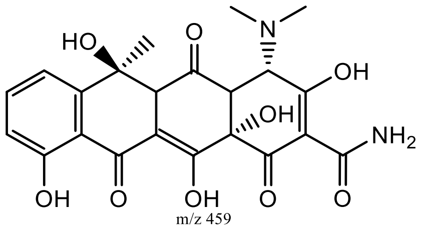 m/z 459	Ora Rat LD50 (mg/kg)<br>Fathead minnow LC50 (96 hr) (mg/L)<br>Daphnia magna LC50 (48hr) (mg/L)	1959.20<br>0.25<br>16.69
**P VII**	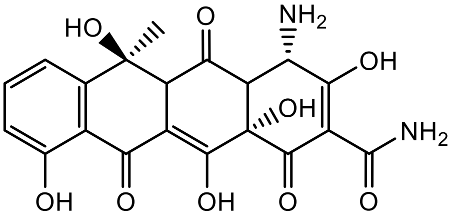 m/z 431	Ora Rat LD50 (mg/kg)<br>Fathead minnow LC50 (96 hr) (mg/L)<br>Daphnia magna LC50 (48hr) (mg/L)	2023.61<br>0.15<br>11.08

References

[1] H. Yu, D. Wang, B. Zhao, Y. Lu, X. Wang, S. Zhu, W. Qin, M. Huo, Enhanced photocatalytic degradation of tetracycline under visible light by using a ternary photocatalyst of $Ag_3PO_4/AgBr/g\text{-}C_3N_4$ with dual Z-scheme heterojunction, Sep. Purif. Technol., 237 (2020) 116365.

[2] L.-X. Yang, J.-C.E. Yang, M.-L. Fu, Magnetic $CoFe_2O_4$ nanocrystals derived from MIL-101 (Fe/Co) for peroxymonosulfate activation toward degradation of chloramphenicol, Chemosphere, 272 (2021) 129567.

[3] B. Liu, W. Song, H. Wu, Y. Xu, Y. Sun, Y. Yu, H. Zheng, S. Wan, Enhanced oxidative degradation of norfloxacin using peroxymonosulfate activated by oily sludge carbon-based nanoparticles $CoFe_2O_4$/OSC, Chem. Eng. J., 400 (2020) 125947.

[4] X. Wang, J. Jiang, Y. Ma, Y. Song, T. Li, S. Dong, Tetracycline hydrochloride degradation over manganese cobaltate ($MnCo_2O_4$) modified ultrathin graphitic carbon nitride (g-C_3N_4) nanosheet through the highly efficient activation of peroxymonosulfate under visible light irradiation, J. Colloid. Interface Sci., 600 (2021) 449-462.

[5] J. Jiang, X. Wang, C. Yue, S. Liu, Y. Lin, T. Xie, S. Dong, Efficient photoactivation of peroxymonosulfate by Z-scheme nitrogen-defect-rich $NiCo_2O_4$/g-C_3N_4 for rapid emerging pollutants degradation, J. Hazard. Mater., 414 (2021) 125528.
